# The Completeness of Intervention Descriptions in Randomised Trials of Supervised Exercise Training in Peripheral Arterial Disease

**DOI:** 10.1371/journal.pone.0150869

**Published:** 2016-03-03

**Authors:** Garry A. Tew, Sally Brabyn, Liz Cook, Emily Peckham

**Affiliations:** 1 Department of Sport, Exercise and Rehabilitation, Northumbria University, Newcastle Upon Tyne, United Kingdom; 2 Mental Health and Addiction Group, Department of Health Sciences, University of York, York, United Kingdom; 3 York Trials Unit, Department of Health Sciences, University of York, York, United Kingdom; Nagoya University, JAPAN

## Abstract

Research supports the use of supervised exercise training as a primary therapy for improving the functional status of people with peripheral arterial disease (PAD). Several reviews have focused on reporting the outcomes of exercise interventions, but none have critically examined the quality of intervention reporting. Adequate reporting of the exercise protocols used in randomised controlled trials (RCTs) is central to interpreting study findings and translating effective interventions into practice. The purpose of this review was to evaluate the completeness of intervention descriptions in RCTs of supervised exercise training in people with PAD. A systematic search strategy was used to identify relevant trials published until June 2015. Intervention description completeness in the main trial publication was assessed using the Template for Intervention Description and Replication checklist. Missing intervention details were then sought from additional published material and by emailing authors. Fifty-eight trials were included, reporting on 76 interventions. Within publications, none of the interventions were sufficiently described for all of the items required for replication; this increased to 24 (32%) after contacting authors. Although programme duration, and session frequency and duration were well-reported in publications, complete descriptions of the equipment used, intervention provider, and number of participants per session were missing for three quarters or more of interventions (missing for 75%, 93% and 80% of interventions, respectively). Furthermore, 20%, 24% and 26% of interventions were not sufficiently described for the mode of exercise, intensity of exercise, and tailoring/progression, respectively. Information on intervention adherence/fidelity was also frequently missing: attendance rates were adequately described for 29 (38%) interventions, whereas sufficient detail about the intensity of exercise performed was presented for only 8 (11%) interventions. Important intervention details are commonly missing for supervised exercise programmes in the PAD trial literature. This has implications for the interpretation of outcome data, the investigation of dose-response effects, and the replication of protocols in future studies and clinical practice. Researchers should be mindful of intervention reporting guidelines when attempting to publish information about supervised exercise programmes, regardless of the population being studied.

## Introduction

Peripheral arterial disease (PAD) is a manifestation of systemic atherosclerosis in which one or more branches of the lower aorta become partially or completely occluded, impeding blood flow to the lower extremities. Its prevalence increases from around 8–10% at age 60–69 years to >15% in adults aged >80 years, and it has been estimated that >200 million people worldwide have PAD [1]. The clinical spectrum of PAD is broad; at the milder end individuals may be asymptomatic or they may experience exertion-induced leg pain/discomfort (including intermittent claudication and atypical leg symptoms), and at the severe end individuals have limb-threatening ischaemia (critical limb ischaemia) associated with rest pain and ulceration/gangrene, which may lead to lower-extremity amputation. Functional impairment and functional decline are common in PAD, even among those who are asymptomatic [2]. PAD is also associated with an increased risk of myocardial infarction, ischaemic stroke, heart failure, and vascular death reflecting the systemic atherosclerotic burden [3].

Exercise training has been an extensively-studied strategy for improving the functional status of individuals with PAD. For example, a recent Cochrane review including 1,816 participants across 30 randomised controlled trials concluded that exercise training improved walking ability by 50% to 200% compared with usual care or placebo in individuals with intermittent claudication [4]. Another recent systematic review and meta-analysis involving 2,074 participants across 27 studies demonstrated that supervised exercise improves maximum walking distance to a greater extent than unsupervised exercise (effect size at 12 months = 0.56, 95% confidence intervals 0.34 to 0.77) [5]. Other studies have evaluated the benefits of exercise in PAD patients without claudication [6,7], the effects of exercise on haemodynamic, functional and quality of life outcomes [8–10], and the effectiveness of exercise versus alternative treatments such as lower-limb revascularisation [11,12], intermittent pneumatic compression [13], and pharmacological therapy [14–16]. This body of research has culminated in supervised exercise programmes being recommended as a first-line therapy for symptomatic PAD in clinical guidelines around the world [17–19]. However, it is currently unclear how to best tailor exercise programmes throughout the PAD continuum (e.g. in asymptomatic disease, stable claudication, or after revascularisation) or to achieve a particular outcome (e.g. to increase or maintain walking capacity, or reduce cardiovascular risk), and this is reflected in the broad guidelines on exercise for intermittent claudication provided by the United Kingdom’s National Institute for Health and Care Excellence: “2 hours of supervised exercise a week for a 3-month period encouraging people to exercise to the point of maximal pain.” [20]

A critical yet sometimes overlooked feature of exercise-related research is the reporting of the exercise intervention. Exercise programmes comprise several components all of which interplay to determine the overall training response. These include the mode and intensity of exercise, the duration and frequency of exercise sessions, and the duration of the programme. Inadequate reporting of these components, and other important information such as the actual dose of exercise received, can limit the interpretation of study findings and the translation of research evidence into clinical practice. Previous work has highlighted deficiencies in the reporting of a range of non-pharmacological interventions in published trials [21]. A recent review of exercise-based cardiac rehabilitation trials has also reported that only 8% of interventions sufficiently described all items required for replication within the main publication, increasing to 15% after reviewing additional published material, and 43% after contacting trial authors [22]. This latter study used the recently-developed Template for Intervention Description and Replication (TIDieR) checklist and guide [23], which provides a structure for assessing the completeness of intervention descriptions.

The aim of this study was to examine the completeness of reporting of supervised exercise programmes for people with PAD in published trials using the TIDieR checklist. We also assessed if incomplete intervention descriptions could be improved by reviewing additional published material and contacting trial authors.

## Methods

### Eligibility criteria

The sample of trials was identified via a systematic search for publications reporting on the effects of supervised exercise programmes on functional and quality of life outcomes for PAD. Studies were included if they were randomised controlled trials, published in English, comparing any supervised exercise programme versus control or any other exercise (e.g. unsupervised exercise programme), medical or surgical intervention. Participants must have had clinically-diagnosed lower-limb PAD, which was either asymptomatic or associated with claudication/exertional leg symptoms of any severity. Trials that included patients with critical limb ischaemia were excluded. Studies must have reported at least one of the following outcome measures to be included: treadmill walking performance (i.e. pain-free or maximum walking distance or time), 6-minute walking distance, or health-related quality of life. For inclusion, an exercise programme needed to be described as supervised or, if a mixture supervised and unsupervised exercise was used, the start of the programme should have comprised a least four weeks of supervised training involving at least one supervised session per week. The exercise programme may have been provided to participants in any setting (e.g. hospital, community centre). Interventions including other components (e.g. dietary modification, smoking cessation, or revascularisation) were included; however, this review focussed solely on the completeness of reporting of the supervised exercise component. Trials in which all groups were offered the same supervised exercise programme with or without some other intervention component(s) (e.g. exercise + drug versus exercise alone) were excluded. Where multiple supervised exercise programmes were investigated within a single study, each exercise arm was considered a separate intervention.

### Search strategy

A systematic search of multiple electronic databases (EMBASE, MEDLINE, Cochrane Central Register of Controlled Trials) was performed and included publications up to June 2015. The search strategy of Lauret *et al*. was used [24], and is presented in S1 Search Strategy. This included a combination of relevant free-text and MeSH terms for the population and intervention, with methodological filters to limit results to randomised controlled trials, systematic reviews, and meta-analyses. Reference lists of included studies, existing systematic reviews and meta-analyses were also searched to identify trials eligible for inclusion.

After removing duplicates, titles and abstracts were examined for potential relevance. The first 10% of titles and abstracts were screened independently by two reviewers (SB and EP). As this showed excellent agreement, the remaining 90% were screened by one reviewer only (GT, SB, LC or EP). Full-text screening was performed independently by two reviewers (GT and either SB, LC or EP) using a purpose-built screening form, and any concerns about study eligibility were resolved through discussion or through consultation with a third reviewer.

### Assessment of intervention description

Intervention descriptions in eligible trials were assessed using the TIDieR checklist (Table 1), which contains guidance on the reporting of 12 intervention items [23]. Items 1 and 2 capture the intervention name and rationale. Items 3 through 9 cover the core procedural and contextual elements of the intervention required for replication. Item 3 –What: Materials–was only marked as complete if the make and model of the equipment used was described or, in the case of treadmill exercise, if a statement was provided about whether a motorised or non-motorised treadmill was used. Item 4 –What: Procedures–was only marked as complete if the mode(s) and structure of the exercise sessions were sufficiently described to allow replication. In the case of walking exercise, it needed to be clear whether treadmill or overground walking was used and whether participants walked on the flat or on a gradient. For Item 5 –Intervention Provider–we required a clear description of who supervised the exercise sessions, including what specific training they received in delivering the intervention, and a description of their expertise and background. For Item 6 –Mode of Delivery–all sessions were supervised, so we only required information about whether participants trained one-to-one with supervisors or in groups, as well as the maximum number of participants per session if group training was used. Item 7 was about the location(s) where the intervention occurred. As this study was specifically concerned with exercise interventions, Item 8 –When and How Much–was assessed in its component parts to include the essential elements of the exercise dose: intensity of exercise, session frequency, session length, and overall intervention duration [22]. Item 9 –Tailoring–required a clear description of how the exercise programme was individualised and progressed. The final items (10–12) record modifications to, and fidelity of, the intervention. Item 10 –Modifications–refers to modifications that occurred to the intervention at a study level after recruitment had commenced. We considered Item 11 –How Well: Planned–as comprising 2 parts: the first was about if any strategies, besides direct supervision, were used to maintain or improve intervention fidelity; the second was about what procedures were used to assess intervention adherence or fidelity, such as exercise logbooks and heart rate monitoring. Finally, Item 12 –How Well: Actual–required authors to describe the extent to which the delivered intervention varied from the intended intervention, for example, through the provision of data about how many exercise sessions were completed, and the duration and intensity of those sessions.

**Table 1:** Brief description of the Template for Intervention Description and Replication (TIDieR) items that were used to assess intervention reporting (adapted from refs [22] and [23]).

Item no.	Item name	Item description
1	Brief name	A name or a phrase which describes the intervention
2	Why	Describe the rationale, theory, or goal of the elements essential to the intervention
3	What: materials	Describe any physical or informational materials used in the intervention, including the make and model of exercise equipment and what materials were provided to participants or used in intervention delivery or in training of intervention providers
4	What: procedures	Describe each of the procedures, activities, and/or processes used in the intervention, including any enabling or support activities
5	Provider	Describes the intervention provider(s) and their expertise, background, and any specific training given
6	How	Describe whether the supervised exercise programme was delivered individually or in a group; if group, then state the maximum number of participants per session
7	Where	Describe the type(s) of location(s) where the intervention occurred, including any necessary infrastructure or relevant features
8	When and how much	Describes the dose/schedule of the intervention including the following:
	(a) Intensity	The intensity of exercise used in the intervention (e.g., target severity of claudication pain during walking)
	(b) Frequency	The frequency of exercise sessions
	(c) Session time	The duration of each individual exercise session
	(d) Overall duration	The overall duration of the supervised exercise programme
9	Tailoring	If the intervention was planned to be personalised, titrated or adapted, then describe what, why, when and how
10	Modifications	Describes any modifications to the intervention during the course of the study
11	How well: planned	
	(a) fidelity strategies	Describe any strategies, besides direct supervision, which were used to maintain or improve intervention fidelity
	(b) fidelity assessment	Describe what procedures were used to assess intervention adherence or fidelity, e.g., exercise logbooks
12	How well: actual	Describe the extent to which the delivered intervention varied from the intended intervention, e.g., through the provision of data about how many exercise sessions were completed, and the duration and intensity of those sessions

Each intervention in the included trials was appraised for completeness of reporting of each checklist item. Items missing from the intervention description, or not described in sufficient detail for replication, were considered to be incomplete. For each intervention that was incompletely described, a list of missing items was generated.

### Collection of further intervention details

For each trial, reference lists, as well as citation and author tracking, were used to determine whether additional information about the intervention had been published in other sources. If relevant publications were found, these were retrieved and relevant data extracted.

If no further sources describing the intervention could be located, or if some items were still incomplete, attempts were made to contact the authors of the trials for further information. For more recent studies, email addresses were generally available within the article. Contacting authors of older studies involved searching for their most recent publications and accompanying contact details, using Google, or workplace staff directories. Corresponding authors were emailed questions specifically related to the missing intervention information. Authors were sent up to 3 emails, each a fortnight apart. If emails were not delivered because of an incorrect or inactive address, attempts were made to contact co-authors. If authors responded with the requested information, the completeness of the TIDieR checklist was reassessed.

### Data analysis

A modified version of the TIDieR checklist [23] was used as a data extraction form for compiling details about each checklist item. An Excel spreadsheet was used to track the completion of missing items, including searches for additional sources and follow-up with email contacts. Data extraction from all included trials was completed independently by two reviewers (GT and EP). Disagreements were resolved through discussion. Data were analysed using descriptive statistics, as described previously [22].

## Results

**Fig 1:**
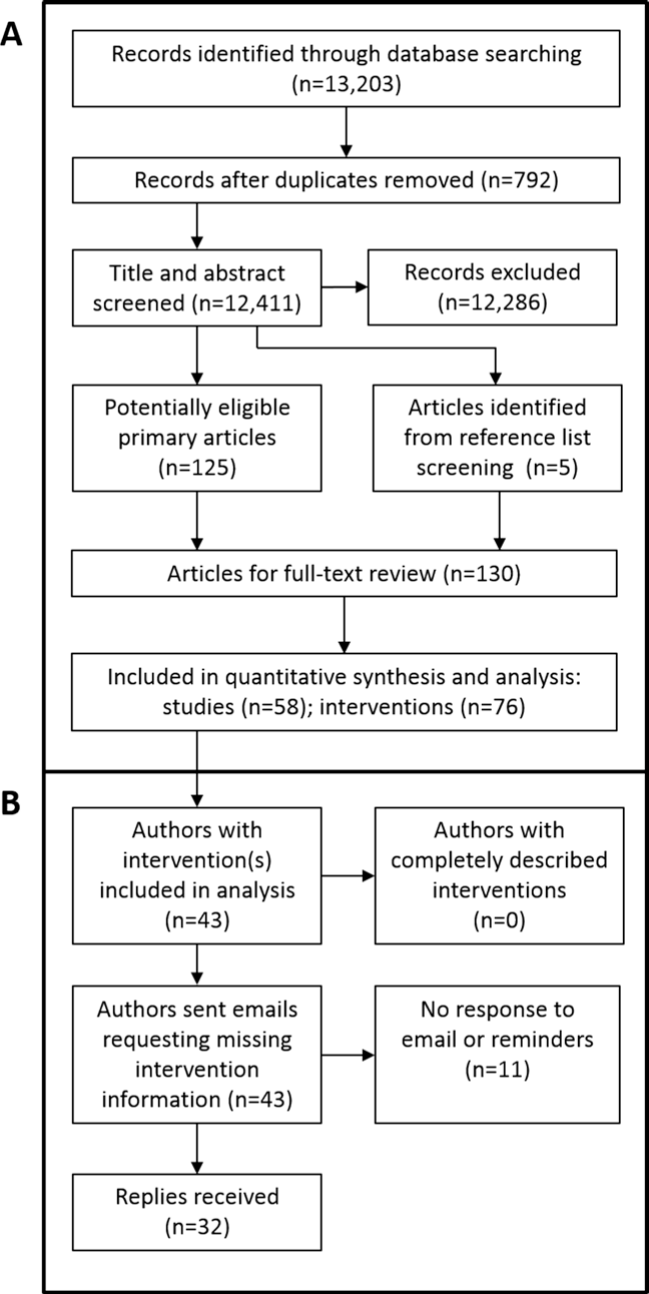
The flow of studies through the trial selection process (A) and the author contact process (B).

After de-duplication, abstract screening, and full-text review, the final sample comprised 58 trials reporting on 76 interventions (Fig 1A). A list of the included trials, including a brief description of the intervention(s) and comparator(s), is provided in S1 Table. The most common study design was a supervised exercise programme versus some type of control condition (N = 21, 36%). Six trials (10%) evaluated an exercise programme initiated after revascularisation; in all other cases, the intervention was provided to participants who were being managed conservatively. One trial included individuals with PAD who were either asymptomatic or had exertion leg symptoms not consistent with intermittent claudication [6]. Another study included people with PAD with or without claudication [7]. All other trials limited recruitment to people with intermittent claudication. The trials were published between 1973 and 2014; however 37 (64%) were published within the last 10 years. Across the 58 trials, 43 corresponding authors were listed (Fig 1B). Eight authors had published two or more trials eligible for inclusion in the analysis.

The types of exercise interventions used are briefly described in S1 Table. Fifty-one interventions (67%) involved aerobic training, 8 (11%) involved resistance training, and 12 (16%) involved a combination of aerobic and resistance training. The specific type of training used was unclear in the remaining 5 (7%) interventions. Of the 63 aerobic training components (excluding any warm-up or cool-down exercises), 37 (59%) were based solely on treadmill or overground walking, 2 (3%) involved walking with poles, 4 (6%) involved arm-crank exercise, 3 (5%) involved cycle ergometer exercise, 1 involved StairMaster exercise, 1 involved plantarflexion exercise (four 4-min intervals), and 15 (24%) involved a combination of 2 or more aerobic exercise modalities (circuit training is included in this category). Of the 20 resistance training components, 6 (30%) included only lower-body exercises, 1 included only upper-body exercises, and 9 (45%) included a combination of upper- and lower-body exercises. The specific resistance exercises used was unclear in the remaining 4 (20%) cases. The duration of supervised exercise training ranged from 2 weeks [14,16] to 18 months [25].

### Description of supervised exercise programmes in main publications

**Fig 2:**
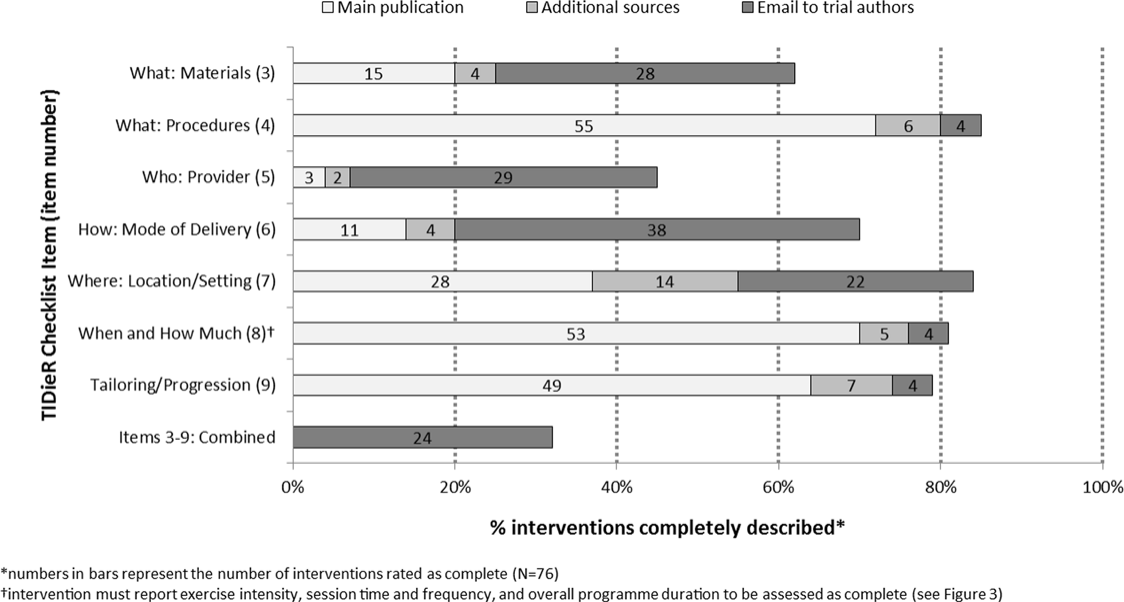
For each Template for Intervention Description and Replication (TIDieR) item, the percentage of interventions which completely reported the item in the main trial publication, after reviewing additional published sources, and after contact with trial authors.

**Fig 3:**
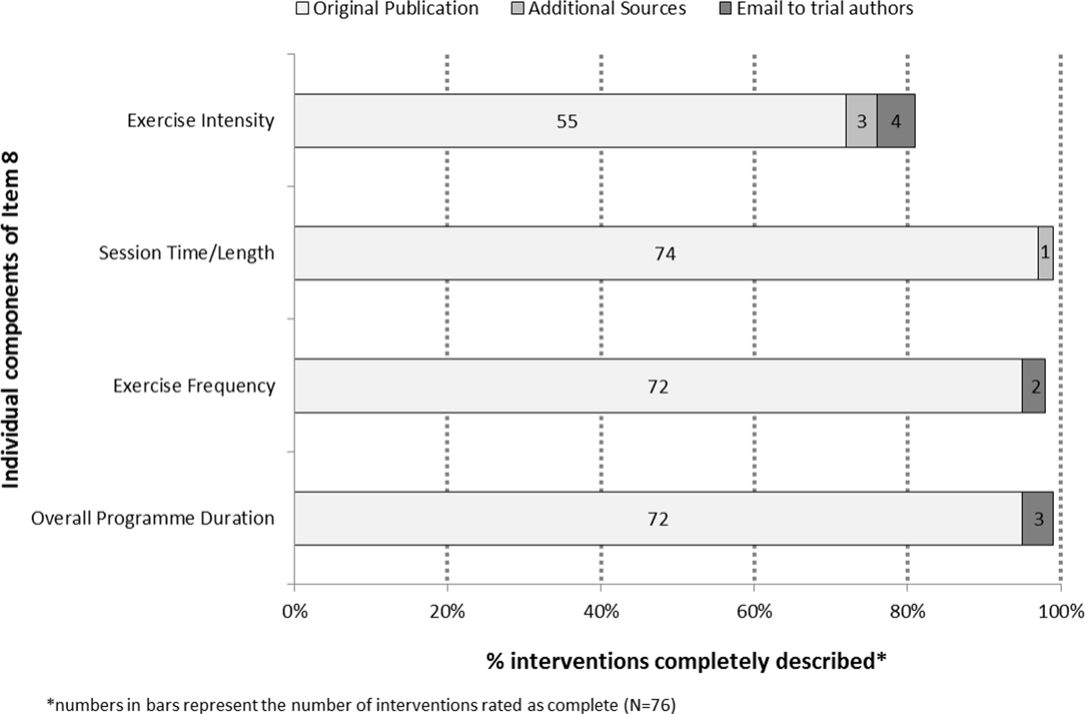
Percentage of interventions which completely reported each component of Item 8 (the When and How Much of exercise) in the main trial publication, after reviewing additional published sources and after contact with trial authors.

A brief intervention description (Item 1) and rationale (Item 2) were found in the main trial publication for all 76 interventions. Fig 2 displays the number and percentage of interventions for which checklist items 3 through 9 were assessed as complete. None of the main publications provided complete information for all of these core items; however, three of the items were reasonably well reported: 72% of interventions were assessed as complete for procedures (Item 4), 70% for the when and how much of the programme (Item 8), and 64% for intervention tailoring/progression (Item 9). Of the 21 interventions that were inadequately described for procedures, all were lacking some information regarding the mode(s) of exercise used, and 13 were also missing information about the order of exercises or proportion of time devoted to each exercise. In terms of the component parts of the when and how much item (Item 8; Fig 3), programme duration, session frequency and session duration were complete for ≥95% of interventions, whereas the intensity of exercise prescribed was missing or unclear for 21 (28%) interventions. Items that were poorly described included materials (Item 3; 20%), provider (Item 5; 4%), mode of delivery (Item 6; 14%), and setting (Item 7; 37%).

Only one main publication described a modification to a supervised exercise programme after recruitment had commenced (Item 10): Parmenter and colleagues explained that the leg press exercise was dropped from both of their resistance training programmes after one participant had experienced exacerbation of a heel fissure while using it [26]. Only 7 interventions (9%), across 5 publications [12,27–30], reported on planned strategies to enhance intervention fidelity besides direct supervision (Item 11). These strategies included use of an intervention oversight committee [12], an educational component promoting regular exercise [28,29], and allowing participants to make up missed sessions [27]. Procedures for assessing adherence or fidelity were described for only 31 (41%) interventions. Furthermore, data on the extent to which the intervention was delivered as planned (Item 12) was provided for only 25 (33%) interventions. Such data was most commonly limited to the number/percentage of sessions attended (11 of 25 interventions).

### Description of supervised exercise programmes after reviewing additional published material

Additional sources of information were found for 39 (67%) of the 58 included studies; these are listed in S1 Table. The reviewing of these additional sources did not lead to any of the interventions becoming completed for items 3 through 9. Improvements in individual item descriptions were most common for location (Item 7), tailoring/progression (Item 9), and procedures (Item 4); complete for 14, 7 and 6 interventions, respectively (Fig 2). Only one additional intervention modification (Item 10) was identified: Langbein and colleagues explained that their exercise programme duration was changed from 12 to 24 weeks part way through their study [31]. Procedures for assessing adherence or fidelity (Item 11) were described for a further 3 interventions, taking the total number of interventions satisfying this item to 34 (45%). Information on intervention adherence/fidelity (Item 12) continued to be frequently missing: attendance rates were now adequately described for 29 (38%) interventions, whereas data on the actual intensity of exercise performed was now known for only 8 (11%) interventions.

### Description of supervised exercise programmes after emailing trial authors

All 43 corresponding authors needed to be contacted for additional information about missing intervention details. Email addresses were obtained for all authors. Thirty-two authors (74%) responded and provided additional information, whereas 11 (26%) did not, despite two reminder emails being sent (Fig 1B). Contacts with authors resulted in the completion of all Items 3 to 9 for 24 interventions (32%). The responses also provided a complete description of materials (Item 3) for 28 interventions, provider (Item 5) for 29 interventions, mode of delivery (Item 6) for 38 interventions, and setting (Item 7) for 22 interventions (Fig 2). Procedures for assessing adherence or fidelity (Item 11) and information on participant compliance (Item 12) were also described for a further 16 and 11 interventions, respectively.

## Discussion

This study has revealed that none of the interventions used in trials of supervised exercise training in PAD were described in sufficient detail to allow full replication. The interventions were reported well for some criteria, such as procedures and when and how much (with 80% and 76% having an adequate description, respectively), but very poorly on other criteria such as materials, provider, and number of participants per session (25%, 7% and 20%, respectively). The proportion of interventions that were sufficiently-described to allow full replication increased to 32% after emailing trial authors; however, this process was time-consuming and 26% of authors did not respond. Procedures for assessing adherence or fidelity were missing for over half of the interventions, and the reporting of adherence/fidelity data was worse, with information on the intensity of exercise performed presented for only 11% of interventions.

The completeness of descriptions of exercise-based interventions has not been widely studied. Three previous reviews, one in the area of stroke rehabilitation [32], and two in cancer survivorship [33,34], have evaluated the reporting of exercise programmes according to the FITT principle, which is a well-established core methodology in exercise prescription that stands for the Frequency, Intensity, Time and Type of exercise. Using the TIDieR checklist, the type of exercise is captured in Item 4 (Procedures), whereas frequency, intensity and time are captured in our adapted version of Item 8 (When and How Much). In the present study, the frequency and time (i.e. duration) of sessions were well-reported components of FITT (both ≥95%). The intensity of exercise was the most frequently missing component (24%), and this was also the case for the three other reviews: stroke, 40% [32]; breast cancer, 21% [33]; other cancer, 30% [34]. Sufficient detail regarding the mode of exercise was also missing for 17% of the PAD interventions, which is exactly the same proportion as for the breast cancer review [33], but worse than that reported in the stroke and other-cancer reviews (5% and 6%, respectively) [32,34]. Mode and intensity of exercise are key determinants of training response, and a clear understanding of these components (and others) is critical for interpreting study findings, the investigation of dose-response effects, and the replication of protocols in future studies and clinical practice. Although we found that missing information can often be obtained by contacting authors directly, this process has several limitations, the main ones being difficulty in locating current email addresses when authors’ contact details had changed, and authors not being able to access or recall intervention details, which was a particular problem for some of the older trials. Also, a substantial proportion of authors (26%) did not respond to emails even after two reminders; a non-response rate that is comparable to that of others attempting to contact authors via email for additional information [22].

The previous review of Abell *et al*. probably provides the most directly-comparable data to that of the present study, because that study examined the reporting quality of exercise-based cardiac rehabilitation interventions using the TIDieR checklist [22]. These authors similarly reported that intensity of exercise was the most frequently incomplete component of FITT (35%), and a very low proportion of interventions were sufficiently described for all items required for replication (Items 3–9): 8% based on the main trial publication, increasing to 15% after reviewing additional published material, and 43% after contacting trial authors. However, their results did differ markedly to ours on several of the individual checklist items. For example, a lower proportion of interventions were adequately described for provider and location than in the cardiac rehabilitation literature (7% and 55% versus 65% and 91%, respectively). The reasons for this are unclear, but we acknowledge the possibility that we may have been harsher than Abell and colleagues in our assessments of these checklist items (see Strengths and Limitations for further discussion), rather than authors of PAD trials being generally worse at reporting specific intervention details. Irrespective of these differences, the overall conclusions of both studies are the same in that important intervention details are commonly unreported.

Perhaps one of the most striking findings of this review was the extremely poor completion rates for Items 11 and 12, which deal with reporting on the planned versus the delivered intervention. To illustrate, after reviewing all published material relating to the included studies, procedures for assessing adherence or fidelity were described for less than half (47%) of the interventions. Furthermore, relatively few studies described adherence to the planned intervention, with only 38% providing attendance rates and 11% providing intensity data. Simply stating exercise session attendance rates alone does not reveal the exact intensity and duration of exercise that was completed which have implications in relation to observed changes or lack thereof. Poor reporting of adherence to exercise interventions is also a common problem in other areas of exercise medicine. For example, in a review of exercise interventions for stroke survivors, a target for the frequency, intensity, time and type of exercise was described for 95%, 60%, 92% and 95% of interventions, respectively, whereas adherence to these components was reported in only 57%, 14%, 19% and 24% of them [32]. Similar findings have been reported in the cancer literature [33,34].

It should not be assumed that exercise programmes are delivered as planned. Indeed, there are several reasons why they might not be, including patient-related factors (e.g. lack of motivation, claudication pain or other co-morbidities limiting the amount of exercise that can be performed [35,36]), environmental factors (e.g., lack of transport limiting regular attendance) and health concerns (e.g., avoidance of claudication pain due to fear that it may cause damage [35]). Studies that have used more detailed fidelity assessments have also shown discrepancies between what was planned and completed. For example, in a fidelity evaluation of a 10-week high-intensity interval training programme for 17 adolescents, Taylor *et al*. reported that the median (interquartile range) proportion of exercise intervals meeting their high-intensity criterion of ≥90% of individual maximal heart rate was 58% (42% to 68%) [37]. Their method of evaluating intervention fidelity may have been useful in many of the PAD exercise trials which used interval training protocols; researchers could have quantified the average number and duration of intervals completed, the average intensity of each interval as measured using a claudication pain scale, rating of perceived exertion and/or heart rate, and the within- and between-subject variability in these measures. We recommend that authors should follow methods such as this to allow an accurate interpretation of study outcomes. We also recommend that authors should follow the TIDieR guide [23] when writing both their protocol and trial report. Although it may only be possible to provide key intervention details in a trial’s primary paper because of journal word limit restrictions, there are several places where additional information can be made accessible, including trial websites, protocol papers, and online supplementary material.

### Strengths and limitations

A strength of this study was that the externally-generated and tested criteria of the TIDieR checklist were applied to evaluate the completeness of intervention descriptions in all PAD exercise trials. A systematic search strategy was used to identify eligible trials for inclusion, with no restrictions on the type of journal or year of publication. However, we did exclude trials that were not written in English. Also, while we recognise that randomised controlled trials are not the only source of evidence for the value of supervised exercise programmes in PAD, they are often considered the “gold standard” by which treatment effectiveness is evaluated. Therefore, we decided to focus this review on reports of randomised trials. Other strengths of this review include duplicate rating and inclusion of a process for obtaining missing intervention details from authors.

A limitation of this review relates to the type of data that is collected by the TIDieR checklist. While all the criteria are dichotomous (in that they require a yes or no response), the justification behind this categorisation has different degrees of interpretation. This could have resulted in overly harsh assessments of completeness for certain criteria. For example, some researchers may have thought that “treadmill” would have been a sufficient description for a treadmill walking programme to receive a “yes” response for Item 3 (Materials), whereas we thought that the make and model of the treadmill should be stated, or it at least be stated whether a motorised or non-motorised system was used. The TIDieR guide [23] is not explicitly clear on this point, and several of the other criteria are also open to interpretation. Nevertheless, we have clearly described how we used the checklist and two researchers had to agree on the item categorisation before the final results were generated. A further limitation of this study was that we did not apply the checklist to the comparator group(s) of the included trials. The reporting of control/comparator conditions has been shown to be poor [38], and the problem of labelling as usual or standard care with no further explanation deserves further emphasis.

## Conclusion

We have demonstrated that important intervention details are commonly missing for supervised exercise programmes in the PAD trial literature. The omission of essential information about interventions has been described as a substantial, yet remediable, contributor to the enormous worldwide waste in research funding [39]. Indeed, it has implications for the interpretation of outcome data, the investigation of dose-response effects, and the replication of protocols in future studies and clinical practice. Researchers should be mindful of intervention reporting guidelines when attempting to publish information about supervised exercise programmes, regardless of the population being studied. The findings of this study are helpful in highlighting the specific areas where intervention descriptions can be improved.

## Supporting Information

S1 Dataset Study data. (XLSX)

S1 Search Strategy Search strategies for electronic databases. (DOCX)

S1 Table Details of the included trials. (DOCX)
