# Supplementary material for: The Completeness of Intervention Descriptions in Randomised Trials of Supervised Exercise Training in Peripheral Arterial Disease
Source: PLoS One. 2016 Mar 3;11(3):e0150869. doi: 10.1371/journal.pone.0150869 (PMC4777572; doi:10.1371/journal.pone.0150869)
Supplement: S1 Search Strategy — (DOCX) [file pone.0150869.s002.docx]

**S1 Search Strategy. Search strategies for electronic databases.**

**Medline Search Strategy**

1. exp Intermittent Claudication/

2. exp Peripheral Arterial Disease/

3. Peripheral Vascular Diseases/

4. (peripheral obstructive art* or iliac art* or femoropop* or claudic$ or dysvascular$ or peripheral vascular disease or leg isch? mia).ab,ti.

5. ((arter$ or vascu$ or peripher$) adj5 (obstruct$ or occlus$ or steno$ or isch?em*)).ab,ti.

6. or/1-5

7. exp Exercise Therapy/

8. exp Exercise/

9. (sport* or walk* or exercise* or rehabil$ or training$ or activit$).ab,ti.

10. or/7-9

11. randomized controlled trial.pt.

12. controlled clinical trial.pt.

13. randomized.ab.

14. randomly.ab.

15. (trial or groups).ab.

16. or/11-15

17. exp animals/ not humans.sh.

18. 16 not 17

19. 6 and 10 and 18

**Records Identified = 2458**

**Embase Search Strategy**

1. exp peripheral occlusive artery disease/

2. peripheral vascular disease.mp. [mp=title, abstract, heading word, drug trade name, original title, device manufacturer, drug manufacturer, device trade name, keyword]

3. leg ischemia.mp. [mp=title, abstract, heading word, drug trade name, original title, device manufacturer, drug manufacturer, device trade name, keyword]

4. exp iliac artery obstruction/

5. (peripheral obstructive art* or iliac art* or femoropop* or claudic$ or dysvascular$ or peripheral vascular disease or leg isch? mia).ab,ti.

6. ((arter$ or vascu$ or peripher$) adj5 (obstruct$ or occlus$ or steno$ or isch?em*)).ab,ti.

7. 1 or 2 or 3 or 4 or 5 or 6

8. exp kinesiotherapy/

9. exp exercise/

10. (sport* or walk* or exercise* or rehabil$ or training$ or activit$).ab,ti.

11. 8 or 9 or 10

12. (random$ or factorial$).ab,ti.

13. ((doubl? or singl?) adj1 blind$).ab,ti.

14. assign$.ab,ti.

15. allocat$.ab,ti.

16. double-blind procedure/

17. randomized controlled trial/

18. single-blind procedure/

19. 12 or 13 or 14 or 15 or 16 or 17 or 18

20. 7 and 11 and 19

21. exp ANIMAL/ or NONHUMAN/ or exp ANIMAL EXPERIMENT/

22. exp HUMAN/

23. 21 not 22

24. 20 not 23

**Records Identified = 1442**

**CENTRAL Search Strategy**

#1 MeSH descriptor: [Arteriosclerosis] this term only

#2 MeSH descriptor: [Arteriolosclerosis] this term only

#3 MeSH descriptor: [Arteriosclerosis Obliterans] this term only

#4 MeSH descriptor: [Atherosclerosis] this term only

#5 MeSH descriptor: [Arterial Occlusive Diseases] this term only

#6 MeSH descriptor: [Intermittent Claudication] this term only

#7 MeSH descriptor: [Ischemia] this term only

#8 MeSH descriptor: [Peripheral Vascular Diseases] explode all trees

#9 atherosclero* or arteriosclero* or PVD or PAOD or PAD

#10 (arter* or vascular or vein* or veno* or peripher*) near (occlus* or reocclus* or re-occlus* or steno* or obstruct* or lesio* or block* or harden* or stiffen*)

#11 peripheral near/3 dis*

#12 claudic* or IC

#13 isch*

#14 #1 or #2 or #3 or #4 or #5 or #6 or #7 or #8 or #9 or #10 or #11 or #12 or #13

#15 MeSH descriptor: [Exercise] explode all trees

#16 MeSH descriptor: [Physical Therapy Modalities] explode all trees

#17 MeSH descriptor: [Physical Exertion] this term only

#18 MeSH descriptor: [Sports] explode all trees

#19 MeSH descriptor: [Leisure Activities] this term only

#20 MeSH descriptor: [Fitness Centers] this term only

#21 MeSH descriptor: [Physical Exertion] this term only

#22 (physical near/3 (exercise* or exertion or endurance or therap* or conditioning or activit* or fitness or train*)):ti,ab,kw

#23 (exercise near/3 (train* or intervention* or protocol* or program* or therap or activit* or regim*)):ti,ab,kw

#24 (fitness near/3 (train* or intervention* or protocol* or program* or therap or activit* or regim* or centre* or center*)):ti,ab,kw

#25 ((training or conditioning) near/3 (circuit or intervention* or protocol* or program* or activit* or regim*)):ti,ab,kw

#26 (walk* or run* or treadmill or aerobic or swim* or danc* or weight or squat* or lunge or bend* or raise or cycling or step):ti,ab,kw

#27 kinesiotherap*:ti,ab,kw

#28 ((endurance or aerobic or cardio*) near/3 (fitness or train* or intervention* or protoco* or program* or therap* or activit* or regim*)):ti,ab,kw

#29 #15 or #16 or #17 or #18 or #19 or #20 or #21 or #22 or #23 or #24 or #25 or #26 or #27 or #28

#30 #14 and #29 in Trials

**Records Identified = 8511**
