# Supplementary material for: The Completeness of Intervention Descriptions in Randomised Trials of Supervised Exercise Training in Peripheral Arterial Disease
Source: PLoS One. 2016 Mar 3;11(3):e0150869. doi: 10.1371/journal.pone.0150869 (PMC4777572; doi:10.1371/journal.pone.0150869)
Supplement: S1 Table — (DOCX) [file pone.0150869.s003.docx]

**S1 Table. Details of the included trials.**

| **Main publication** | **Additional information** | **Supervised exercise programme(s)** | **Comparator(s)** |
| --- | --- | --- | --- |
| Allen JD, Stabler T, Kenjale A, et al. Plasma nitrite flux predicts exercise performance in peripheral arterial disease after 3 months of exercise training. Free Radic Biol Med 2010; 49:1138–1144. | Duscha BD, Robbins JL, Jones WS, et al. Angiogenesis in skeletal muscle precede improvements in peak oxygen uptake in peripheral artery Disease patients. Arterioscler Thromb Vasc Biol 2011; 31:2742–2748.  Jones SW, Duscha BD, Robbins JL, et al. Alteration in angiogenic and anti-angiogenic forms of vascular endothelial growth factor-A in skeletal muscle of patients with intermittent claudication following exercise training. Vasc Med 2012; 17:94–100.  Allen JD, Stabler T, Kenjale A, et al. Diabetes status differentiates endothelial function and plasma nitrite response to exercise stress in peripheral arterial disease following supervised training. J Diabetes Complications 2014;28:219–225. | Aerobic – intermittent treadmill walking exercise to moderately severe claudication pain | Unsupervised exercise programme |
| Arosio E, Cuzzolin L, De Marchi S, et al. Increased endogenous nitric oxide production induced by physical exercise in peripheral arterial occlusive disease patients. Life Sci 1999;65:2815–22. | Arosio E, Minuz P, Prior M, et al. Vascular adhesion molecule-1 and markers of platelet function before and after a treatment with iloprost or a supervised physical exercise program in patients with peripheral arterial disease. Life Sci 2001;69:421–33. | Aerobic and resistance – treadmill walking, cycle ergometer exercise and muscular strengthening of the legs | Daily iloprost infusion |
| Badger SA, Soong CV, O'Donnell ME, et al. Benefits of a supervised exercise program after lower limb bypass surgery. Vasc Endovascular Surg 2007;41:27–32. | *None found* | Aerobic – treadmill walking exercise (4-10 weeks post-revascularisation) | Unsupervised exercise programme and best medical therapy |
| Bø E, Hisdal J, Cvancarova M, et al. Twelve-months follow-up of supervised exercise after percutaneous transluminal angioplasty for intermittent claudication: a randomised clinical trial. Int J Environ Res Public Health 2013;10:5998–6014. | Bø E, Bergland A, Stranden E, et al. Effects of 12 Weeks of Supervised Exercise After Endovascular Treatment: A Randomized Clinical Trial. Physiother Res Int 2014; doi: 10.1002/pri.1608.  Nilsson BB, Hellesnes B, Westheim A, et al. Group-based aerobic interval training in patients with chronic heart failure: Norwegian Ullevaal Model. Phys Ther 2008;88:523–35. | Aerobic and resistance – aerobic interval training involving simple dance movements and walking, as well as calisthenics (initiated after percutaneous transluminal angioplasty for 12 weeks) | Usual care |
| Cheetham DR, Burgess L, Ellis M, et al. Does supervised exercise offer adjuvant benefit over exercise advice alone for the treatment of intermittent claudication? A randomised trial. Eur J Vasc Endovasc Surg 2004;27:17–23. | *None found* | Aerobic and resistance – circuit training involving walking, step climbing and lower-limb strengthening exercises | Exercise advice and best medical therapy |
| Collins EG, Langbein WE, Orebaugh C, et al. PoleStriding exercise and vitamin E for management of peripheral vascular disease. Med Sci Sports Exerc 2003;35:384–93. | Langbein WE, Collins EG, Orebaugh C, et al. Increasing exercise tolerance of persons limited by claudication pain using polestriding. J Vasc Surg 2002;35:887–93.  Collins EG, Langbein WE, Orebaugh C, et al. Cardiovascular training effect associated with polestriding exercise in patients with peripheral arterial disease. J Cardiovasc Nurs 2005;20:177–85. | Aerobic – Treadmill or overground walking exercise using Exerstrider™ poles with Vitamin E supplementation or placebo (considered as one exercise intervention) | Vitamin E supplementation or placebo |
| Collins EG, OʼConnell S, McBurney C, et al. Comparison of walking with poles and traditional walking for peripheral arterial disease rehabilitation. J Cardiopulm Rehabil Prev 2012;32:210-8. | Collins EG, McBurney C, Butler J, et al. The Effects of Walking or Walking-with-Poles Training on Tissue Oxygenation in Patients with Peripheral Arterial Disease. Int J Vasc Med 2012;2012:985025.  Trial registration: ClinicalTrials.gov  NCT00719355 | (1) Aerobic – Treadmill or overground walking exercise using Exerstrider™ poles  (2) Aerobic – Treadmill or overground walking exercise (without poles) | *None listed here as study compared two supervised exercise programmes* |
| Crowther RG, Spinks WL, Leicht AS, et al. Effects of a long-term exercise program on lower limb mobility, physiological responses, walking performance, and physical activity levels in patients with peripheral arterial disease. J Vasc Surg 2008;47:303–9. | Crowther RG, Spinks WL, Leicht AS, et al. The influence of a long term exercise program on lower limb movement variability and walking performance in patients with peripheral arterial disease. Hum Mov Sci 2009;28:494–503.  Leicht AS, Crowther RG, Golledge J. Influence of peripheral arterial disease and supervised walking on heart rate variability. J Vasc Surg 2011;54:1352–9.  Crowther RG, Leicht AS, Spinks WL, et al. Effects of a 6-month exercise program pilot study on walking economy, peak physiological characteristics, and walking performance in patients with peripheral arterial disease. Vasc Health Risk Manag 2012;8:225–32.  Leicht AS, Crowther RG, Golledge J. Influence of regular exercise on body fat and eating patterns of patients with intermittent claudication. Int J Mol Sci 2015;16:11339–54. | Aerobic – Treadmill walking exercise | Usual care |
| Dahllöf AG, Björntorp P, Holm J, et al. Metabolic activity of skeletal muscle in patients with peripheral arterial insufficiency.  Eur J Clin Invest 1974;4:9–15. | *None found* | ? – “ dynamic leg exercises” | Placebo tablets |
| Delaney CL, Miller MD, Chataway TK, et al. A randomised controlled trial of supervised exercise regimens and their impact on walking performance, skeletal muscle mass and calpain activity in patients with intermittent claudication. Eur J Vasc Endovasc Surg 2014;47:304–10. | Delaney CL, Miller MD, Allan RB, et al. The impact of different supervised exercise regimens on endothelial function in patients with intermittent claudication. Vascular. 2014 Nov 18. pii: 1708538114558329.  Delaney CL, Miller MD, Dickinson KM, et al. Change in dietary intake of adults with intermittent claudication undergoing a supervised exercise program and compared to matched controls. Nutr J 2014;13:100.  Trial registration: ClinicalTrials.gov  NCT01871779 | (1) Aerobic – intermittent treadmill walking exercise to the point of unbearable claudication pain  (2) Aerobic and resistance – lower-body resistance exercises and treadmill walking exercise until the onset of claudication pain only | *None listed here as study compared two supervised exercise programmes* |
| Gardner AW, Katzel LI, Sorkin JD, et al. Exercise rehabilitation improves functional outcomes and peripheral circulation in patients with intermittent claudication: a randomized controlled trial. J Am Geriatr Soc 2001;49:755–62. | Gardner AW, Katzel LI, Sorkin JD, et al. Effects of long-term exercise rehabilitation on claudication distances in patients with peripheral arterial disease: a randomized controlled trial. J Cardiopulm Rehabil 2002;22:192–8.  Killewich LA, Macko RF, Montgomery PS, et al. Exercise training enhances endogenous fibrinolysis in peripheral arterial disease. J Vasc Surg 2004;40:741–5. | Aerobic – intermittent treadmill walking exercise to near-maximal claudication pain | Usual care |
| Gardner AW, Montgomery PS, Flinn WR, et al. The effect of exercise intensity on the response to exercise rehabilitation in patients with intermittent claudication. J Vasc Surg 2005;42:702–9. | Trial registration: ClinicalTrials.gov  NCT00654810 | (1) Aerobic – treadmill walking exercise at 40% of maximal exercise capacity  (2) Aerobic – treadmill walking exercise at 80% of maximal exercise capacity | *None listed here as study compared two supervised exercise programmes* |
| Gardner AW, Parker DE, Montgomery PS, et al. Efficacy of quantified home-based exercise and supervised exercise in patients with intermittent claudication: a randomized controlled trial. Circulation 2011;123:491–8. | Gardner AW, Parker DE, Montgomery PS, et al. Diabetic women are poor responders to exercise rehabilitation in the treatment of claudication. J Vasc Surg 2014;59:1036–43.  Trial registration: ClinicalTrials.gov  NCT00618670 | Aerobic – treadmill walking exercise at 40% of maximal exercise capacity | (1) Unsupervised exercise programme  (2) Usual care |
| Gardner AW, Montgomery PS, Parker DE. Optimal exercise program length for patients with claudication. J Vasc Surg 2012;55:1346–54. | *None found* | Aerobic – intermittent treadmill walking exercise to near-maximal claudication pain | Usual care |
| Gardner AW, Parker DE, Montgomery PS, et al. Step-monitored home exercise improves ambulation, vascular function, and inflammation in symptomatic patients with peripheral artery disease: a randomized controlled trial. J Am Heart Assoc 2014;3:e001107. | Mauer K, Gardner AW, Dasari TW, et al. Clot strength is negatively associated with ambulatory function in patients with peripheral artery disease and intermittent claudication. Angiology 2015;66:354–9. | (1) Aerobic – treadmill walking exercise at 40% of maximal exercise capacity  (2) Resistance – Attention control – low-volume, low-intensity whole-body resistance training | Unsupervised exercise programme |
| Gelin J, Jivegård L, Taft C, et al. Treatment efficacy of intermittent claudication by surgical intervention, supervised physical exercise training compared to no treatment in unselected randomised patients I: one year results of functional and physiological improvements. Eur J Vasc Endovasc Surg 2001;22:107–13. | Taft C, Karlsson J, Gelin J, et al. Treatment efficacy of intermittent claudication by invasive therapy, supervised physical exercise training compared to no treatment in unselected randomised patients II: one-year results of health-related quality of life. Eur J Vasc Endovasc Surg 2001;22:114–23.  Taft C, Sullivan M, Lundholm K, et al. Predictors of treatment outcome in intermittent claudication. Eur J Vasc Endovasc Surg 2004;27:24–32. | Aerobic – “specific walking training” | (1) Endovascular or open surgical revascularisation  (2) Usual care |
| Guidon M, McGee H. One-year effect of a supervised exercise programme on functional capacity and quality of life in peripheral arterial disease. Disabil Rehabil 2013;35:397–404. | Guidon M, McGee H. Recruitment to clinical trials of exercise: challenges in the peripheral arterial disease population.  Physiotherapy 2013;99:305–10.  Trial registration: ISRCTN73659272 | Aerobic – exercise at 70-80% maximum exercise capacity or 70-80% predicted maximum heart rate on treadmill, stepper, elliptical trainer, recumbent cycle ergometer, upper/lower-limb cycle ergometer | Usual care |
| Hiatt WR, Regensteiner JG, Hargarten ME, et al. Benefit of exercise conditioning for patients with peripheral arterial disease. Circulation 1990;81:602–9. | *None found* | Aerobic – intermittent treadmill walking exercise to moderately severe claudication pain | Usual activity |
| Hiatt WR, Wolfel EE, Meier RH, et al. Superiority of treadmill walking exercise versus strength training for patients with peripheral arterial disease. Implications for the mechanism of the training response. Circulation 1994;90:1866–74. | Hiatt WR, Regensteiner JG, Wolfel EE, et al. Effect of exercise training on skeletal muscle histology and metabolism in peripheral arterial disease. J Appl Physiol 1996;81:780–8.  Regensteiner JG, Steiner JF, Hiatt WR. Exercise training improves functional status in patients with peripheral arterial disease. J Vasc Surg 1996;23:104–15. | (1) Aerobic – intermittent treadmill walking exercise to moderate claudication pain  (2) Resistance – lower-body resistance exercises | Usual activity |
| Hobbs SD, Marshall T, Fegan C, et al. The constitutive procoagulant and hypofibrinolytic state in patients with intermittent claudication due to infrainguinal disease significantly improves with percutaneous transluminal balloon angioplasty. J Vasc Surg 2006;43:40–6. | Hobbs SD, Bradbury AW. The EXercise versus Angioplasty in Claudication Trial (EXACT): reasons for recruitment failure and the implications for research into and treatment of intermittent claudication. J Vasc Surg 2006;44:432–3.  Hobbs SD, Marshall T, Fegan C, et al.  The effect of supervised exercise and cilostazol on coagulation and fibrinolysis in intermittent claudication: a randomized controlled trial. J Vasc Surg 2007;45:65–70.  Trial registration: ISRCTN90757358 | Aerobic and resistance – circuit training involving walking and upper- and lower-limb strengthening exercises | (1) Best medical therapy and percutaneous transluminal balloon angioplasty  (2) Best medical therapy |
| Holm J, Dahllöf AG, Björntorp P, et al. Enzyme studies in muscles of patients with intermittent claudication. Effect of training. Scand J Clin Lab Invest Suppl 1973;128:201–5. | *None found* | ? – “ dynamic leg exercises” | Placebo tablets |
| Jakubsevičienė E, Vasiliauskas D, Velička L, et al. Effectiveness of a new exercise program after lower limb arterial blood flow surgery in patients with peripheral arterial disease: a randomized clinical trial. Int J Environ Res Public Health 2014;11:7961–76. | Jakubsevičienė E, Vasiliauskas D, Kubilius R, et al. Exercise-based rehabilitation improves  hemodynamic responses after lower limb  arterial blood flow surgery. Brit J Med Med Res 2014; 4(10): 2089–99. | (1) Aerobic and resistance – aerobic exercise at 60-85% maximum heart rate on treadmill, stepper, and ergometers, and exercise on “resistance devices”  (2) Aerobic – track walking, stair climbing and treadmill exercise at 60-85% maximum heart rate  (both interventions lasted 18 days and were initiated on average 7 days after femoral-popliteal artery bypass grafting) | *None listed here as study compared two supervised exercise programmes* |
| Jones PP, Skinner JS, Smith LK, et al. Functional improvements following StairMaster vs. treadmill exercise training for patients with intermittent claudication.  J Cardiopulm Rehabil 1996;16:47–55. | *None found* | (1) Aerobic – intermittent StairMaster exercise to severe claudication pain  (2) Aerobic – intermittent treadmill walking exercise to severe claudication pain | *None listed here as study compared two supervised exercise programmes* |
| Kakkos SK, Geroulakos G, Nicolaides AN. Improvement of the walking ability in intermittent claudication due to superficial femoral artery occlusion with supervised exercise and pneumatic foot and calf compression: a randomised controlled trial. Eur J Vasc Endovasc Surg 2005;30:164–75. | *None found* | Aerobic – intermittent treadmill walking exercise to moderately severe claudication pain | (1) Intermittent foot and calf pneumatic compression therapy  (2) Exercise advice |
| Kruidenier LM, Nicolaï SP, Rouwet EV, et al. Additional supervised exercise therapy after a percutaneous vascular intervention for peripheral arterial disease: a randomized clinical trial. J Vasc Interv Radiol 2011;22:961–8. | Willigendael EM, Bendermacher BL, van der Berg C, et al. The development and implementation of a regional network of physiotherapists for exercise therapy in patients with peripheral arterial disease, a preliminary report. BMC Health Serv Res 2005;5:49.  Kruidenier LM, Nicolaï SP, Hendriks EJ, et al. Supervised exercise therapy for intermittent claudication in daily practice. J Vasc Surg 2009;49:363–70.  Trial registration: ClinicalTrials.gov  NCT00497445 | Aerobic and resistance – based mostly on intermittent treadmill walking exercise to near maximal claudication pain, but also included cycle ergometer training and resistance exercises (initiated 3 weeks after percutaneous vascular intervention) | Percutaneous vascular intervention |
| Lundgren F, Dahllöf AG, Lundholm K, et al. Intermittent claudication--surgical reconstruction or physical training? A prospective randomized trial of treatment efficiency. Ann Surg 1989;209:346–55. | Lundgren F, Dahllöf AG, Scherstén T, et al. Muscle enzyme adaptation in patients with peripheral arterial insufficiency: spontaneous adaptation, effect of different treatments and consequences on walking performance. Clin Sci 1989;77:485–93. | ? – “dynamic leg exercises” with or without reconstructive arterial surgery (considered as one exercise intervention; exercise was initiated 6 weeks after the last operation in those receiving surgery) | Reconstructive surgery |
| Maejima Y, Yasu T, Ueba H, et al. Exercise after heparin administration: new therapeutic program for patients with-option arteriosclerosis oblitrans. Circ J 2005;69:1099–104. | *None found* | Aerobic – intermittent walking exercise with or without daily intravenous heparin injections (considered as one exercise intervention) | Daily intravenous heparin injections |
| Mannarino E, Pasqualini L, Innocente S, et al. Physical training and antiplatelet treatment in stage II peripheral arterial occlusive disease: alone or combined? Angiology 1991;42:513–21. | Mannarino E, Pasqualini L, Menna M, et al. Effects of physical training on peripheral vascular disease: a controlled study. Angiology 1989;40:5–10. | Aerobic and resistance - walking, hopping, jogging and lower-limb resistance exercises with or without dipyridamole 75 mg three times daily and aspirin 330 mg once daily (considered as one exercise intervention) | Dipyridamole 75 mg three times daily and aspirin 330 mg once daily |
| Mazari FA, Khan JA, Carradice D, et al. Randomized clinical trial of percutaneous transluminal angioplasty, supervised exercise and combined treatment for intermittent claudication due to femoropopliteal arterial disease. Br J Surg 2012;99:39–48. | Lee HL, Mehta T, Ray B, et al. A non-randomised controlled trial of the clinical and cost effectiveness of a supervised exercise programme for claudication. Eur J Vasc Endovasc Surg 2007;33:202–7.  Mazari FA, Gulati S, Rahman MN, et al. Early outcomes from a randomized, controlled trial of supervised exercise, angioplasty, and combined therapy in intermittent claudication. Ann Vasc Surg 2010;24:69–79.  Mazari FA, Khan JA, Carradice D, et al. Economic analysis of a randomized trial of percutaneous angioplasty, supervised exercise or combined treatment for intermittent claudication due to femoropopliteal arterial disease. Br J Surg 2013;100:1172–9.  Trial registration: ClinicalTrials.gov  NCT00798850 | Aerobic and resistance – circuit training involving walking, cycling, stepping and upper- and lower-limb strengthening exercises with or without percutaneous transluminal angioplasty (considered as one exercise intervention; exercise was initiated 1 week after the operation in those receiving PTA) | Percutaneous transluminal angioplasty |
| McDermott MM, Tiukinhoy S, Greenland P, et al. A pilot exercise intervention to improve lower extremity functioning in peripheral arterial disease unaccompanied by intermittent claudication. J Cardiopulm Rehabil 2004;24:187–96. | *None found* | Aerobic – treadmill walking exercise at a light-to-somewhat hard intensity (11-12 on Borg 6-20 exertion scale) | Usual care |
| McDermott MM, Ades P, Guralnik JM, et al. Treadmill exercise and resistance training in patients with peripheral arterial disease with and without intermittent claudication: a randomized controlled trial. JAMA 2009;301:165–74. | McDermott MM, Domanchuk K, Dyer A, et al. Recruiting participants with peripheral arterial disease for clinical trials: experience from the Study to Improve Leg Circulation (SILC). J Vasc Surg 2009;49:653–659.  Trial registration: ClinicalTrials.gov  NCT00106327 | (1) Aerobic - treadmill walking exercise at a somewhat hard intensity (12-14 on Borg 6-20 exertion scale) if asymptomatic or to near maximal leg symptoms if symptomatic  (2) Resistance – lower-body resistance exercises at a somewhat hard intensity (12-14 on Borg 6-20 exertion scale) | Nutritional information sessions |
| McGuigan MR, Bronks R, Newton RU, et al. Resistance training in patients with peripheral arterial disease: effects on myosin isoforms, fiber type distribution, and capillary supply to skeletal muscle. J Gerontol A Biol Sci Med Sci 2001;56:B302–10. | *None found* | Resistance – periodized programme involving upper- and lower-limb exercises | “non-exercising control” |
| Mika P, Spodaryk K, Cencora A. Effects of treadmill training on walking distance and lower limb blood flow in patients with intermittent claudication. Med Rehab 2005;9:3–9. | Mika P, Spodaryk K, Cencora A, et al. Experimental model of pain-free treadmill training in patients with claudication. Am J Phys Med Rehabil 2005;84:756–62. | Aerobic – intermittent treadmill walking exercise to 85% of pain-free walking  distance | Usual activity |
| Mika P, Spodaryk K, Cencora A, et al. Red blood cell deformability in patients with claudication after pain-free treadmill training. Clin J Sport Med 2006;16:335–40. | *None found* | Aerobic – intermittent treadmill walking exercise to 85% of pain-free walking  distance | Usual activity |
| Mika P, Wilk B, Mika A, et al. The effect of pain-free treadmill training on fibrinogen, haematocrit, and lipid profile in patients with claudication. Eur J Cardiovasc Prev Rehabil 2011;18:754–60. | *None found* | Aerobic – intermittent treadmill walking exercise to the onset of claudication pain | Usual activity |
| Mika P, Konik A, Januszek R, et al. Comparison of two treadmill training programs on walking ability and endothelial function in intermittent claudication. Int J Cardiol 2013;168:838–42. | *None found* | (1) Aerobic – intermittent treadmill walking exercise to the onset of claudication pain  (2) Aerobic – intermittent treadmill walking exercise to moderate claudication pain | *None listed here as study compared two supervised exercise programmes* |
| Murphy TP, Cutlip DE, Regensteiner JG, et al. Supervised exercise versus primary stenting for claudication resulting from aortoiliac peripheral artery disease: six-month outcomes from the claudication: exercise versus endoluminal revascularization (CLEVER) study. Circulation 2012;125:130–9. | Murphy TP, Hirsch AT, Ricotta JJ, et al. The Claudication: Exercise Vs. Endoluminal Revascularization (CLEVER) study: rationale and methods. J Vasc Surg 2008;47:1356–63.  Bronas UG, Hirsch AT, Murphy T, et al. Design of the multicenter standardized supervised exercise training intervention for the claudication: exercise vs endoluminal revascularization (CLEVER) study. Vasc Med 2009;14:313–21.  Murphy TP, Hirsch AT, Cutlip DE, et al. Claudication: exercise vs endoluminal revascularization (CLEVER) study update. J Vasc Surg 2009;50:942–945.  Murphy TP, Reynolds MR, Cohen DJ, et al. Correlation of patient-reported symptom outcomes and treadmill test outcomes after treatment for aortoiliac claudication. J Vasc Interv Radiol 2013;24:1427–35.  Reynolds MR, Apruzzese P, Galper BZ, et al. Cost-effectiveness of supervised exercise, stenting, and optimal medical care for claudication: results from the Claudication: Exercise Versus Endoluminal Revascularization (CLEVER) trial. J Am Heart Assoc 2014;3:e001233.  Murphy TP, Cutlip DE, Regensteiner JG, et al. Supervised exercise, stent revascularization, or medical therapy for claudication due to aortoiliac peripheral artery disease: the CLEVER study. J Am Coll Cardiol 2015;65:999–1009.  Trial registration: ClinicalTrials.gov  NCT00132743 | Aerobic – intermittent treadmill walking exercise to moderate claudication pain | (1) Optimal medical care and stent revascularisation  (2) Optimal medical care |
| Nicolaï SP, Teijink JA, Prins MH et al. Multicenter randomized clinical trial of supervised exercise therapy with or without feedback versus walking advice for intermittent claudication. J Vasc Surg 2010;52:348–55. | Willigendael EM, Bendermacher BL, van der Berg C, et al. The development and implementation of a regional network of physiotherapists for exercise therapy in patients with peripheral arterial disease, a preliminary report. BMC Health Serv Res 2005;5:49.  Kruidenier LM, Nicolaï SP, Hendriks EJ, et al. Supervised exercise therapy for intermittent claudication in daily practice. J Vasc Surg 2009;49:363–70.  Nicolaï SP, Hendriks EJ, Prins MH, et al. Optimizing supervised exercise therapy for patients with intermittent claudication. J Vasc Surg 2010;52:1226–33.  Trial registration: ClinicalTrials.gov  NCT00279994 | Aerobic and resistance – based mostly on intermittent treadmill walking exercise to near maximal claudication pain, but also included cycle ergometer training and resistance exercises | “go home and walk” advice |
| Parmenter BJ, Raymond J, Dinnen P, et al. High-intensity progressive resistance training improves flat-ground walking in older adults with symptomatic peripheral arterial disease. J Am Geriatr Soc 2013;61:1964–70. | Trial registration: ACTRN12609000457246 | (1) Resistance – whole-body, high-intensity, progressive resistance training  (2) Resistance – whole-body, low-intensity, non-progressive resistance training | Usual care |
| Parr BM, Noakes TD, Derman EW. Peripheral arterial disease and intermittent claudication: efficacy of short-term upper body strength training, dynamic exercise training, and advice to exercise at home. S Afr Med J 2009;99:800–4. | *None found* | (1) Aerobic and resistance – intermittent treadmill walking exercise, spin cycling and upper- and lower-body resistance training  (2) Resistance – upper-body resistance exercises | Exercise advice |
| Patterson RB, Pinto B, Marcus B, et al. Value of a supervised exercise program for the therapy of arterial claudication. J Vasc Surg 1997;25:312–8. | Pinto BM, Marcus BH, Patterson RB, et al. On-site versus home exercise programs: Psychological benefits for patients with arterial claudication. J Aging Phys Act 1997;5:311–328.  Braun CM, Colucci AM, Patterson RB.  Components of an optimal exercise program for the treatment of patients with claudication. J Vasc Nurs 1999;17:32–6. | Aerobic and resistance – based mostly on intermittent treadmill walking exercise to near maximal claudication pain, but also included cardiovascular training on rowing, cycle and arm ergometers and “muscle toning” with light hand weights | Unsupervised exercise programme |
| Perkins JM, Collin J, Creasy TS, et al. Exercise training versus angioplasty for stable claudication. Long and medium term results of a prospective, randomised trial. Eur J Vasc Endovasc Surg 1996;11:409–13. | Creasy TS1, McMillan PJ, Fletcher EW, et al. Is percutaneous transluminal angioplasty better than exercise for claudication? Preliminary results from a prospective randomised trial. Eur J Vasc Surg 1990;4:135–40. | Aerobic – a mixture of “dynamic leg exercises” including walking, cycling and stepping | Percutaneous transluminal angioplasty |
| Regensteiner JG, Meyer TJ, Krupski WC, et al. Hospital vs home-based exercise rehabilitation for patients with peripheral arterial occlusive disease. Angiology 1997;48:291–300. | *None found* |  |  |
| Ritti-Dias RM, Wolosker N, de Moraes Forjaz CL, et al. Strength training increases walking tolerance in intermittent claudication patients: randomized trial. J Vasc Surg 2010;51:89–95. | Grizzo Cucato G, de Moraes Forjaz CL, Kanegusuku H, et al. Effects of walking and strength training on resting and exercise cardiovascular responses in patients with intermittent claudication. Vasa 2011;40:390–7.  Menêses AL, de Lima GH, Forjaz CL, et al. Impact of a supervised strength training or walking training over a subsequent unsupervised therapy period on walking capacity in patients with claudication. J Vasc Nurs 2011;29:81–6.  Trial registration: ClinicalTrials.gov  NCT00879697 | (1) Aerobic - treadmill walking exercise at a light-to-somewhat hard intensity (11-13 on Borg 6-20 exertion scale)  (2) Resistance – upper- and lower-body resistance exercises at a light-to-somewhat hard intensity (11-13 on Borg 6-20 exertion scale) | *None listed here as study compared two supervised exercise programmes* |
| Sandercock GR, Hodges LD, Das SK, et al. The impact of short term supervised and home-based walking programmes on heart rate variability in patients with peripheral arterial disease. J Sports Sci Med 2007;6:471–6. | Hodges LD, Sandercock GR, Das SK, et al. Randomized controlled trial of supervised exercise to evaluate changes in cardiac function in patients with peripheral atherosclerotic disease. Clin Physiol Funct Imaging 2008;28:32–7. | Aerobic – treadmill walking exercise at 70-75% peak oxygen uptake | (1) Unsupervised exercise programme  (2) Exercise advice |
| Sanderson B, Askew C, Stewart I, et al. Short-term effects of cycle and treadmill training on exercise tolerance in peripheral arterial disease. J Vasc Surg 2006;44:119–27. | Wood RE, Sanderson BE, Askew CD, et al. Effect of training on the response of plasma vascular endothelial growth factor to exercise in patients with peripheral arterial disease. Clin Sci 2006;111:401–9. | (1) Aerobic – intermittent treadmill walking exercise at 80% peak oxygen uptake  (2) Aerobic – intermittent cycling exercise at 80% peak oxygen uptake | Usual care |
| Savage P, Ricci MA, Lynn M, et al. Effects of home versus supervised exercise for patients with intermittent claudication. J Cardiopulm Rehabil 2001;21:152–7. | *None found* | Aerobic – treadmill walking exercise at 60% of maximal exercise capacity | Unsupervised exercise programme |
| Schlager O, Giurgea A, Schuhfried O, et al. Exercise training increases endothelial progenitor cells and decreases asymmetric dimethylarginine in peripheral arterial disease: a randomized controlled trial. Atherosclerosis 2011;217:240–8. | Schlager O, Hammer A, Giurgea A, et al. Impact of exercise training on inflammation and platelet activation in patients with intermittent claudication. Swiss Med Wkly 2012;142:w13623.    Trial registration: ClinicalTrials.gov  NCT00926081 | Aerobic – intermittent walking exercise to moderate claudication pain | Best medical treatment |
| Slørdahl SA, Wang E, Hoff J, et al. Effective training for patients with intermittent claudication. Scand Cardiovasc J 2005;39:244–9. | *None found* | (1) Aerobic – intermittent treadmill walking exercise at 80% peak oxygen uptake  (2) Aerobic – treadmill walking exercise at 60% peak oxygen uptake | *None listed here as study compared two supervised exercise programmes* |
| Spronk S, Bosch JL, den Hoed PT, et al. Intermittent claudication: clinical effectiveness of endovascular revascularization versus supervised hospital-based exercise training--randomized controlled trial. Radiology 2009;250:586–95. | Fakhry F, Rouwet EV, den Hoed PT, et al. Long-term clinical effectiveness of supervised exercise therapy versus endovascular revascularization for intermittent claudication from a randomized clinical trial. Br J Surg 2013;100:1164–71.  Trial registration: ISRCTN64443682  Trial registration: Netherlands Trial Register NTR199 | Aerobic – treadmill walking exercise to maximum claudication pain | Endovascular revascularisation |
| Stewart AH, Smith FC, Baird RN, et al. Local versus systemic mechanisms underlying supervised exercise training for intermittent claudication. Vasc Endovascular Surg 2008;42:314–20. | *None found* | ? – 5 exercises mainly based on calf muscle | Exercise advice |
| Tew G, Nawaz S, Zwierska I, et al. Limb-specific and cross-transfer effects of arm-crank exercise training in patients with symptomatic peripheral arterial disease. Clin Sci 2009;117:405–13. | Tew GA. Physiological effects of exercise in patients with peripheral vascular disease. PhD Thesis 2009, Sheffield Hallam University.  Trial registration: ISRCTN50394448 | Aerobic – intermittent arm-crank exercise at 60-70% maximum exercise capacity | Usual care |
| Tisi PV, Hulse M, Chulakadabba A, et al. Exercise training for intermittent claudication: does it adversely affect biochemical markers of the exercise-induced inflammatory response? Eur J Vasc Endovasc Surg 1997;14:344–50. | *None found* | ? – active and passive leg exercises performed to the  limit of claudication pain | (1) Percutaneous  transluminal angioplasty  (2) Observation |
| Treat-Jacobson D, Bronas UG, Leon AS. Efficacy of arm-ergometry versus treadmill exercise training to improve walking distance in patients with claudication. Vasc Med 2009;14:203–13. | Bronas UG, Treat-Jacobson D, Leon AS. Comparison of the effect of upper body-ergometry aerobic training vs treadmill training on central cardiorespiratory improvement and walking distance in patients with claudication. J Vasc Surg 2011;53:1557–64.  Trial registration: ClinicalTrials.gov  NCT00895635 | (1) Aerobic – intermittent treadmill walking exercise to moderately severe claudication pain  (2) Aerobic – intermittent arm-crank exercise at 10 watts below maximum watts on preliminary exercise test  (3) Aerobic – a combination of exercise protocol (1) and (2) | Unsupervised exercise programme |
| Tsai JC, Chan P, Wang CH, et al. The effects of exercise training on walking function and perception of health status in elderly patients with peripheral arterial occlusive disease. J Intern Med 2002;252:448–55. | *None found* | Aerobic – treadmill walking exercise at mild-to-moderate claudication pain | Usual care |
| Walker RD, Nawaz S, Wilkinson CH, et al. Influence of upper- and lower-limb exercise training on cardiovascular function and walking distances in patients with intermittent claudication. J Vasc Surg 2000;31:662–9. | Nawaz S, Walker RD, Wilkinson CH, et al. The inflammatory response to upper and lower limb exercise and the effects of exercise training in patients with claudication. J Vasc Surg 2001;33:392–9. | (1) Aerobic – intermittent cycling exercise at the penultimate workload achieved on preliminary exercise test  (2) Aerobic – intermittent arm-crank exercise at the penultimate workload achieved on preliminary exercise test | *None listed here as study only randomised between two supervised exercise programmes* |
| Wang E, Hoff J, Loe H, et al. Plantar flexion: an effective training for peripheral arterial disease. Eur J Appl Physiol 2008;104:749–56. | Helgerud J, Wang E, Mosti MP, et al. Plantar flexion training primes peripheral arterial disease patients for improvements in cardiac function. Eur J Appl Physiol 2009;106:207–15. | Aerobic – individual leg plantar flexion training; four 4-min intervals at 80% of maximal exercise capacity on each leg | Exercise advice |
| Zwierska I, Walker RD, Choksy SA, et al. Upper- vs lower-limb aerobic exercise rehabilitation in patients with symptomatic peripheral arterial disease: a randomized controlled trial. J Vasc Surg 2005;42:1122–30. | Saxton JM, Zwierska I, Hopkinson K, et al. Effect of upper- and lower-limb exercise training on circulating soluble adhesion molecules, hs-CRP and stress proteins in patients with intermittent claudication. Eur J Vasc Endovasc Surg 2008;35:607–13.  Saxton JM, Zwierska I, Blagojevic M, et al. Upper- versus lower-limb aerobic exercise training on health-related quality of life in patients with symptomatic peripheral arterial disease. J Vasc Surg 2011;53:1265–73.  Trial registration: ISRCTN76180797 | (1) Aerobic – intermittent cycling exercise at the penultimate workload achieved on preliminary exercise test  (2) Aerobic – intermittent arm-crank exercise at the penultimate workload achieved on preliminary exercise test | Usual care |
